# Vaccarin alleviates endothelial inflammatory injury in diabetes by mediating miR-570-3p/HDAC1 pathway

**DOI:** 10.3389/fphar.2022.956247

**Published:** 2022-09-01

**Authors:** Taiyue Li, Xiaoyi Yu, Xuerui Zhu, Yuanyuan Wen, Meizhen Zhu, Weiwei Cai, Bao Hou, Fei Xu, Liying Qiu

**Affiliations:** ^1^ Wuxi Medical School, Jiangnan University, Wuxi, Jiangsu, China; ^2^ School of Life Science and Health Engineering, Jiangnan University, Wuxi, Jiangsu, China

**Keywords:** vaccarin, microRNA-570-3p, HDAC1, inflammation, diabetes, endothelial dysfunction

## Abstract

Vaccarin is a flavonoid glycoside, which has a variety of pharmacological properties and plays a protective role in diabetes and its complications, but its mechanism is unclear. In this study, we aim to investigate whether histone deacetylase 1(HDAC1), a gene that plays a pivotal role in regulating eukaryotic gene expression, is the target of miR-570-3p in diabetic vascular endothelium, and the potential molecular mechanism of vaccarin regulating endothelial inflammatory injury through miR-570-3p/HDAC1 pathway. The HFD and streptozotocin (STZ) induced diabetes mice model, a classical type 2 diabetic model, was established. The aorta of diabetic mice displayed a decrease of miR-570-3p, the elevation of HDAC1, and inflammatory injury, which were alleviated by vaccarin. Next, we employed the role of vaccarin in regulating endothelial cells miR-570-3p and HDAC1 under hyperglycemia conditions *in vitro*. We discovered that overexpression of HDAC1 counteracted the inhibitory effect of vaccarin on inflammatory injury in human umbilical vein endothelial cells (HUVECs). Manipulation of miRNA levels in HUVECs was achieved by transfecting cells with miR-570-3p mimic and inhibitor. Overexpression of miR-570-3p could decrease the expression of downstream components of HDAC1 including TNF-α, IL-1β, and malondialdehyde, while increasing GSH-Px activity in HUVECs under hyperglycemic conditions. Nevertheless, such phenomenon was completely reversed by miR-570-3p inhibitor, and administration of miR-570-3p inhibitor could block the inhibition of vaccarin on HDAC1 and inflammatory injury. Luciferase reporter assay confirmed the 3′- UTR of the HDAC1 gene was a direct target of miR-570-3p. In summary, our findings suggest that vaccarin alleviates endothelial inflammatory injury in diabetes by mediating miR-570-3p/HDAC1 pathway. Our study provides a new pathogenic link between deregulation of miRNA expression in the vascular endothelium of diabetes and inflammatory injury and provides new ideas, insights, and choices for the scope of application and medicinal value of vaccarin and some potential biomarkers or targets in diabetic endothelial dysfunction and vascular complications.

## Introduction

Diabetes is a metabolic disease characterized by hyperglycemia due to insulin secretion defects and/or insulin dysfunction (American Diabetes 2014). The incidence rate of diabetes worldwide has increased rapidly in recent years, and it can cause a variety of chronic complications, such as cardiovascular, ophthalmic, nephrotic, and nervous system diseases. It seriously threatens diabetic patients’ health and quality of life. Among them, diabetic angiopathy is one of the most common and serious complications of diabetes, and it is also the main cause of death in patients with type 2 diabetes mellitus (T2DM) (Kannel and McGee 1979; Ostergard and Nyholm et al., 2006). The initial step that leads to T2DM vascular complications is dysfunction and damage of endothelial cells, including endothelial cell apoptosis, inflammation, or anti-angiogenesis induced by hyperglycemia (Yi and Gao 2019).

Endothelial cells are simple squamous cells arranged on the surface of the vascular lumen. They are the interface between circulating blood and vascular intima. Endothelial cells are very important to maintaining a healthy vascular system and are very sensitive the changes in blood glucose levels (Dhananjayan and Koundinya et al., 2016; Vanhoutte and Shimokawa et al., 2017). Under normal circumstances, endothelial cells remain static and regulate vascular tension. In the development of T2DM, hyperglycemia will lead to endothelial cell dysfunction, resulting in endothelial dysfunction (Roberts and Porter 2013). Endothelial dysfunction is the critical first step to the development of diabetic vascular complications, with increased inflammation as a major manifestation (Wu and Jiang et al., 2018). Thus, targeting inflammation is an effective strategy to attenuate diabetes-induced endothelial injury. To date, a causal relationship between inflammation and diabetic angiopathy has been widely accepted, however, the cellular and molecular mechanisms are still not well defined. Nowadays, several inflammation markers are considered to be associated with diabetes (Tang and Wang et al., 2017), such as interleukin-1β (IL-1β) and tumor necrosis factor-α (TNF-α) (Wu and Jiang et al., 2018). Therefore, it is of interest to identify the new factors that may contribute to the pathogenesis (Tang and Wang et al., 2017) and progression of diabetic angiopathy. There is mounting evidence showing that microRNAs (miRNAs) can potentially play a role as biomarkers or intervention targets for endothelial inflammation.

MiRNA (18–23 nt) is a small endogenous non-coding RNA, which participates in post-transcriptional regulation of gene expression (Cheng and Chen et al., 2019). The complementary sites of mature miRNA in 3′UTR bind to the target mRNA, which will lead to the reduction of target mRNA stability and translation (Booton and Lindsay 2014). The abnormal regulation of miRNAs in T2DM has been well confirmed, but its functional role has been rarely studied. We have previously reported that histone deacetylase 1 (HDAC1) is significantly dysregulated in hyperglycemic mouse models and high glucose (HG)-treated endothelial cells (Sun, Cai et al., 2017; Zhu, Lei et al., 2018; Liu, Sun et al., 2020). Using miRNA target prediction sites TargetScan and Miranda (Betel, Koppal et al., 2010; Xie, Cai et al., 2015), we examined a variety of miRNAs and identified miR-570-3p as a potentially novel candidate that may regulate HDAC1. MiR-570-3p has been previously reported in terms of cell proliferation and inflammation (Baker, Vuppusetty et al., 2018), but has not been studied in the context of HG-induced inflammatory injury in endothelial cells.

**FIGURE 1:**
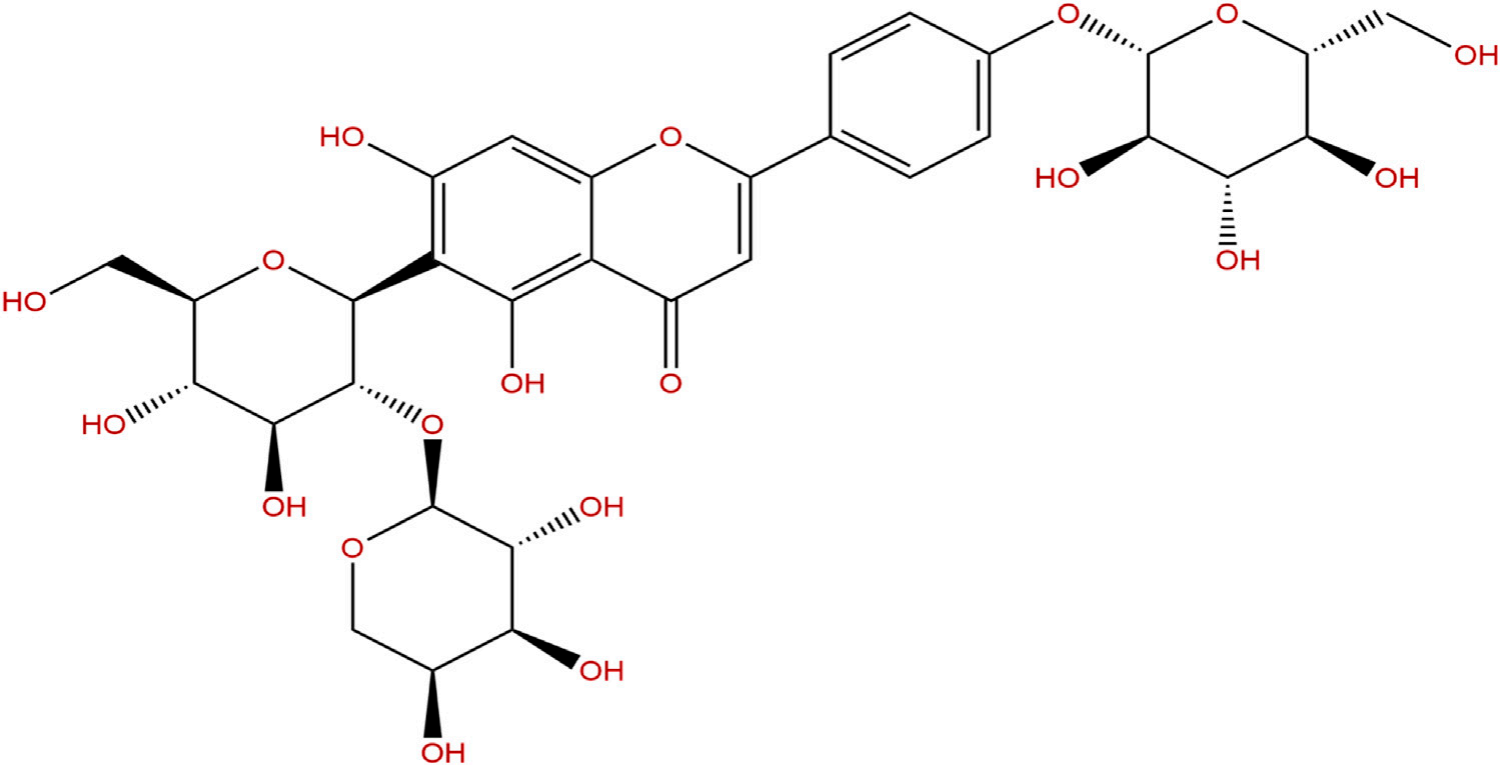
Chemical structure of vaccarin.

Vaccarin (VAC), a flavonoid monomer which contains twelve phenolic hydroxyl groups (Figure 1), is extracted from *Vaccaria segetalis* seeds (Hou et al., 2020; Hou et al., 2020). Vaccarin has a wide range of biological effects, including endothelial cell injury prevention, angiogenesis promotion, wound healing, and liver protection (Sun, Yu et al., 2021). Previous studies have shown that vaccarin can participate in FGF-2 mediated fibroblast growth factor receptor 1 (FGFR-1) induced angiogenesis *in vitro* and *in vivo* (Sun, Cai et al., 2017). In addition, vaccarin can reduce hydrogen peroxide-induced damage to human EA•hy926 endothelial cells by inhibiting the Notch signal (Xie, Cai et al., 2015; Qiu, Du et al., 2016). Recently, it was found that vaccarin can inhibit vascular endothelial dysfunction through ROS/AMPK/miRNA-34a/eNOS signaling pathway through the intervention of the HMEC-1 endothelial dysfunction model induced by HG *in vitro* (Xu, Liu et al., 2019). However, the mechanism of vaccarin on HG-induced inflammatory injury in endothelial cells has not been determined. Therefore, this study reported for the first time that vaccarin inhibited HDAC1 by regulating miR-570-3p to reduce inflammatory injury.

## Materials and methods

### Cell culture and treatments

Human umbilical vein endothelial cells (HUVECs) were provided by the U533 Institute of the French National Institute of Health Medicine (Paris, French) and were cultured in DMEM (HyClone, St. Louis, MO, United States) containing 5 mM glucose, 10% FBS (Lonsera, Shuangru Biotech, Shanghai, China), and 100 U/L penicillin and 100 μg/ml streptomycin (Gibco, Carlsbad, CA, United States). The cells were cultured at 37°C in a humidified incubator of 5% CO_2_ and 95% air. After reaching 70% confluence (Xu, Liu et al., 2019), HUVEC cells were exposed to HG (35 mM) for 24h, then to 5 µM vaccarin or 20 µM metformin (Raj, Natarajan et al., 2021) (Solarbio, Beijing, China) 16 h. Vaccarin was purchased from Shanghai Shifeng Technology (Shanghai, China) (Sun, Yu et al., 2021), purity >98%, the molecular formula is C_32_H_38_O_19_, and molecular mass is 726.63 g/mol.

### Transfection of miRNA mimics and inhibitors

The miR-570-3p mimics, miR-570-3p inhibitor, and corresponding negative controls were synthesized by Gene Pharma (Shanghai, China) and transfected by Lipofectamine^®^ 2000 (Invitrogen, Carlsbad, CA) and cells were exposed to HG (35 mM). The final concentration of the mimics and inhibitor was 50 and 100 nM. The primer sequences for miR-570-3p inhibitor: 5′- GCA AAG GUA AUU GCU GUU UUC G-3’ (Forward); miR-570-3p mimic: 5′-CGA AAA CAG CAA UUA CCU UUG C-3’ (Forward), 5′-AAA GGU AAU UGC UGU UUU CGU U-3’ (Reverse); miRNA-NC inhibitor: 5′- CAG UAC UUU UGU GUA CAA -3’ (Forward); and miRNA-NC mimic: 5′- UUC UCC GAA CGU GUC ACG UTT -3’ (Forward), 5′- ACG UGA CAC GUU CGG AGA ATT -3’ (Reverse).

### Transfection of small interference RNA (siRNA)

The HDAC1 siRNA and corresponding negative controls were synthesized by Gene Pharma (Shanghai, China) and transfected by Lipofectamine^®^ 2000 (Invitrogen, Carlsbad, CA) and cells were exposed to HG (35 mM). The final concentration was 50 nM. The siRNA sequences that targeted HDAC1 were as follows: 5′- GCC GGU CAU GUC CAA AGU ATT -3’ (Forward), 5′- UAC UUU GGA CAU GAC CGG CTT -3’ (Reverse); and siRNA-NC: 5′- UUC UCC GAA CGU GUC ACG UTT -3’ (Forward), 5′- ACG UGA CAC GUU CGG AGA ATT -3’ (Reverse).

### Transfection of plasmids

The pcDNA3.1-HDAC1 plasmids and empty vector pcDNA3.1 were synthesized according to the plasmid synthesis method (Wang, Liu et al., 2013). The HUVEC cells were transfected with Lipofectamine^®^ 2000 (Invitrogen, Carlsbad, CA). The cells were subjected to subsequent operations after transfection for 6 h.

### Real-time quantitative PCR (RT-qPCR)

Total RNA was extracted using Trizol reagent (Cwbio, Beijing, China). MiRNA was extracted using the miRNA Purification Kit (Cwbio, Beijing, China). RNA (1 µg) and miRNA (1 µg) were used to generate cDNA separately using Hifair^®^ III first Strand cDNA Synthesis SuperMix Kit (YESEN, Shanghai, China) and miRNA cDNA Synthesis Kit (Cwbio, Beijing, China). The RT-qPCR was performed by using Hieff UNICON^®^ qPCR SYBR Green Master Mix (YESEN, Shanghai, China) and miRNA qPCR Assay Kit (Cwbio, Beijing, China). 2 −ΔΔCT was used to show the fold change.

### Western blotting

After cell and tissue lysis, the protein concentration was quantified by using the dioctylic acid (BCA) protein detection kit (Beyotime, Shanghai, China), then the protein was denatured at 100 °C for 7 min, and finally stored at -80 °C. Protein lysate was resolved on Tris-glycine SDS-PAGE gels, then electrotransferred onto a PVDF membrane. Antibodies against HDAC1 (1:1000, mouse) (Cell Signaling Technology, Beverly, United States), TNF-α (1:1000, rabbit) (ABclonal, Wuhan, China), IL-1β (1:1000, Rabbit) (ABclonal, Wuhan, China), β-actin (1:5000, mouse) (Proteintech, Wuhan, China) were incubated overnight at 4 °C. Secondary antibodies, anti-rabbit (1:2000) and anti-mouse (1:2000) (Cwbio, Beijing, China) were incubated for 1 h at room temperature (Ai, Li et al., 2021). The blots were visualized by a chemiluminescence detection system (Millipore Darmstadt, Burlingtun, MA, United States). ImageJ (National Institutes of Health, Bethesda, MD, United States) and Image Lab (Bio-Rad, Hercules, CA, United States) were used to semi-quantify bands (Xu, Liu et al., 2019).

### Luciferase reporter assays

A partial HDAC1 mRNA 3′-UTR containing the miR-570-3p target site was constructed into a pGL-3-promoter vector (Promega, Madison, WI). The reporter was co-transfected with a renilla luciferase plasmid driven by a constitutive promoter reporter into cells. Firefly and renilla luciferase luminescence was measured using a Dual-Luciferase reporter kit (Beyotime, Shanghai, China) as the manufacturer’s recommendations. Firefly/renilla ratio was calculated to normalize for variations in transfection efficiencies.

### Determination of indicators related to oxidative stress

The content of malondialdehyde (MDA) was detected by the thiobarbituric acid method, and the activity of glutathione peroxidase (GSH-Px) was determined by colorimetry. Kits were purchased from Nanjing jiancheng (Nanjing, China).

### Animal models and treatments

Experiments were performed on 6 to 8 weeks-old C57BL/6J mice purchased from the Model Animal Research Center of Nanjing University (Nanjing, China). The mice were housed in a light-dark cycle of 12 h in a temperature and humidity-controlled room and treated with free access to clean food and water. The mice were randomly divided into three groups (*n* = 10). One group was treated with a normal diet (14.7 kJ/g, 13% of energy as fat) until the experiments finished (Xu, Liu et al., 2019). The other two groups were fed with an HFD (21.8 kJ/kg, 60% energy as fat, D12492, Research Diets, New Brunswick, NJ, United States) for 4 weeks. After 4 weeks of HFD, fasting for 12 h overnight, streptozotocin (STZ) (Sigma, St. Louis, MO, United States) (120 mg/kg, ip) was injected intraperitoneally to form T2DM. After successful modeling, the three groups of mice received intragastric gavage of either vehicle or vaccarin (1 mg/kg, ig) every day for 6 weeks. Terminal experiments were performed after mice were anesthetized (2–5% isoflurane). The entire aorta was isolated and used for immunohistochemistry, RT-qPCR, western blotting, and vascular function. All protocols were approved by the Experimental Animal Care and Use Committee of Jiangnan University (JN. No20210915c0600129 [339]). The experimental procedures were carried out according to the Guide for the Care and Use of Laboratory Animals published by the US National Institute of Health (NIH publication, eighth edition, 2011) (Xu, Liu et al., 2019).

### Oral glucose tolerance test (OGTT) and insulin tolerance test (ITT)

Mice were fasted for 12 h, then received glucose (2 g/kg, ig) to examine oral glucose tolerance. And mice were fasted for 6 h, then received insulin (0.75 units/kg, ip) to examine insulin tolerance. Blood glucose was measured in veinal blood at 0, 15, 30, 60, and 90 min using a blood glucometer.

### Determination of triglyceride (TG), low-density lipoprotein (LDL), non-esterified fatty acids (NEFA), alanine transaminase (ALT), and aspartate transaminase (AST)

The contents of serum TG, LDL, NEFA, ALT, and AST were detected according to the methods in the corresponding kit (Nanjing Jiancheng).

### Histopathological evaluation

Aortic tissue was fixed with 4% paraformaldehyde and embedded in paraffin. The sections were stained with hematoxylin-eosin (H&E) (Solarbio, Beijing, China) (Sun, Yu et al., 2021) and then observed by optical microscope (Nikon, Japan).

### Immunohistochemistry

Aortic sections were de-paraffinized with xylene, followed by antigen retrieval by heating in citrate buffer (10 mM). The experiment was performed by SP Rabbit and Mouse HRP Kit (Cwbio, Beijing, China) (Tan, Jiang et al., 2022). Sections were probed with appropriate primary antibodies. TNF-α antibody (ABclonal, Wuhan, China), and IL-1β antibody (ABclonal, Wuhan, China) were used at a 1:100 dilution followed by a biotinylated secondary antibody, streptavidin peroxidase solution, DAB peroxidase substrate, and hematoxylin counterstain or by an AlexaFluor^®^647-conjugated anti-mouse IgG (1:500) as the secondary antibody.

### Statistical analysis

All results were defined as mean ± SEM from at least three independent experiments. *t*-test was used for the comparisons between the two groups. For multiple group comparisons, statistical analysis was performed by ANOVA followed by Dunnett’s test. Differences with *p* value <0.05 were regarded as significant (Xu, Liu et al., 2019).

## Results

### Vaccarin can significantly improve the physiological state and blood glucose level of T2DM mice

**FIGURE 2:**
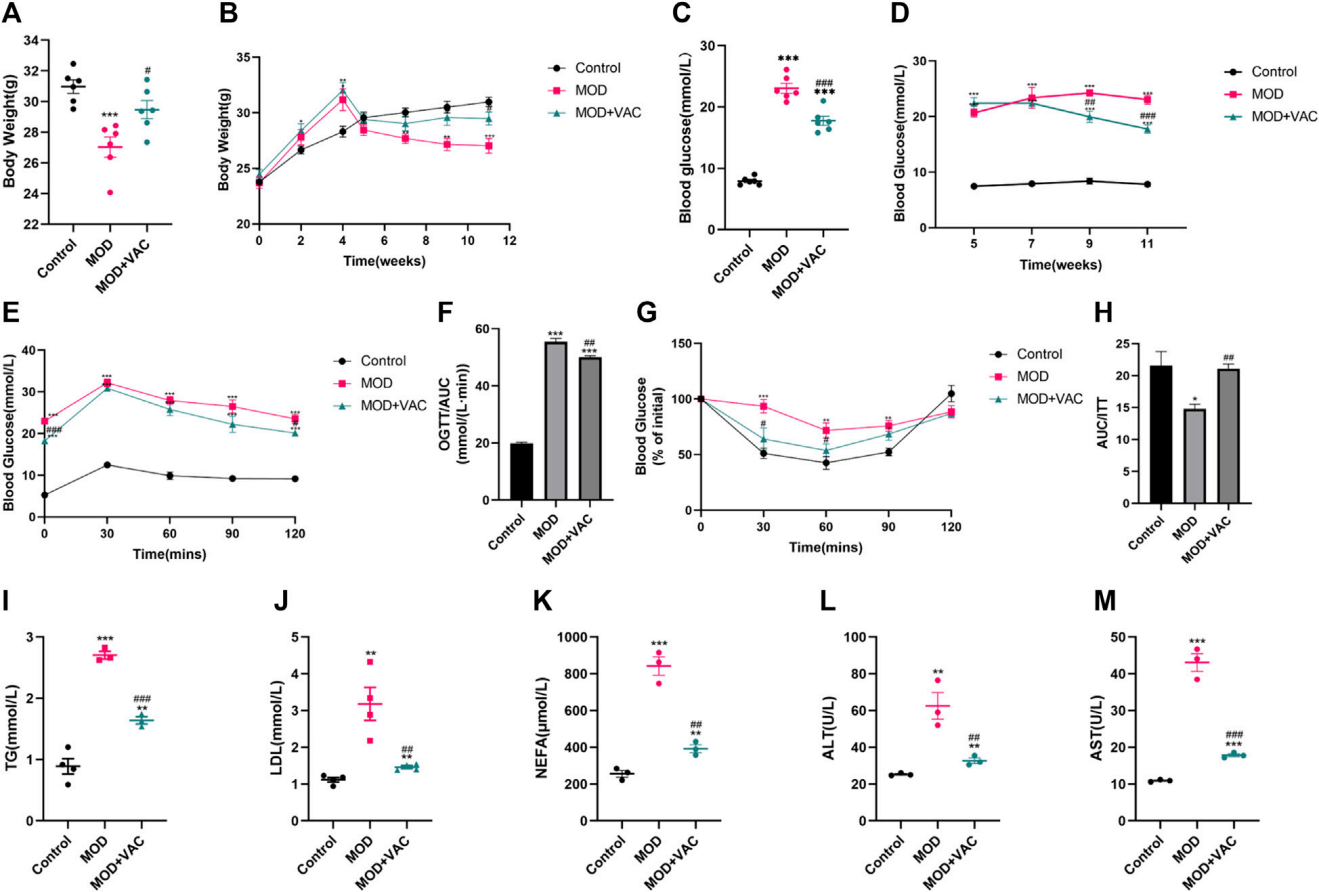
Vaccarin can significantly improve the physiological state and blood glucose level of T2DM mice. **(A)**. The weight of the mice at the terminal of the experiment. (The first 5 weeks were the modeling period and the last 6 weeks were the administration period.) **(B)**. Weekly weight changes after the start of the experiment. **(C)**. Fasting blood glucose. After 6 weeks of treatment, fasting blood glucose was tested in the mice. **(D)**. Weekly changes of fasting blood glucose after successful modeling. **(E)**. Oral glucose tolerance test (OGTT). **(F)**. Area under the curve (AUC) level of OGTT. **(G)**. Insulin tolerance test (ITT). **(H)**. Area under the curve (AUC) level of ITT. **(I–M)**. Content of serum TG, LDL, NEFA, ALT and AST. Values are mean ± SEM. *n* = 3-6 in each group. *p < 0.05, **p < 0.01, ***p < 0.001 vs. Control; ^#^ *p* < 0.05, ^##^ *p* < 0.01, ^###^ *p* < 0.001 vs. MOD.

With the development of T2DM, significantly lower levels of body weight were observed in T2DM mice, but the body weight increased slightly after vaccarin treatment (Figures 2A, B). Fasting blood glucose levels in the vaccarin-treated group decreased steadily and continuously compared with the model group (Figures 2C, D), suggesting that vaccarin partly controlled the elevation of blood glucose in T2DM mice. The results of the OGTT showed that blood glucose level after oral administration of glucose was significantly lower in the vaccarin intervention group than that of the T2DM mice. The results of the ITT showed that insulin resistance appeared in T2DM mice, which could be alleviated by vaccarin (Figures 2E–H). Both the OGTT and ITT results demonstrated that vaccarin not only improved glucose tolerance but also restored the impaired insulin sensitivity in T2DM mice. Compared with the control group, serum levels of lipid metabolism-related indicators TG, LDL, NEFA, ALT, and AST were increased in T2DM mice, while they had improved significantly upon administering vaccarin (Figures 2I–M). These findings suggested that vaccarin could alleviate glycolipid metabolism disorder to a certain extent in T2DM mice.

### Vaccarin improved aortic inflammatory injury in T2DM mice

**FIGURE 3:**
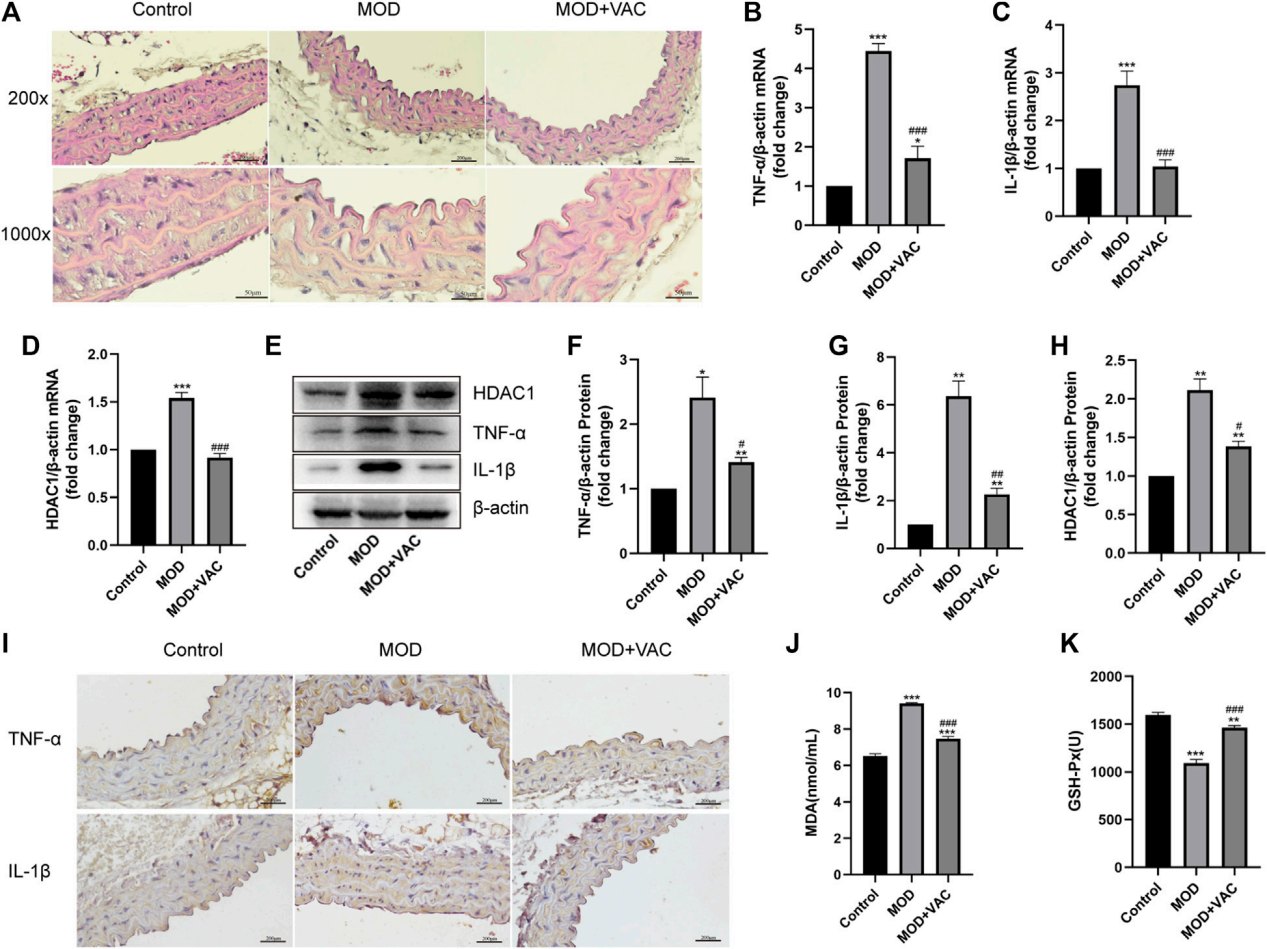
Vaccarin improved aortic inflammatory injury in T2DM mice. **(A)**. Representative photomicrographs of aortic tissue with H&E (200X, 1000X). **(B–D)**. The effects of vaccarin on the TNF-α mRNA, IL-1β mRNA, and HDAC1 mRNA expression in aortic tissue in comparison with T2DM mice. **(E–H)**. Western blotting analysis of HDAC1, TNF-α, and IL-1β protein expression levels in aortic tissue. Gray value of western blotting. **(I)**. Representative photomicrographs of aortic tissue with immunohistochemical staining for TNF-α and IL-1β (200X). **(J)**. Content of serum MDA. **(K)**. Activity of serum GSH-Px. Values are mean ± SEM. *n* = 3 in each group. *p < 0.05, **p < 0.01, ***p < 0.001 vs. Control; ^#^ *p* < 0.05, ^##^ *p* < 0.01, ^###^ *p* < 0.001 vs. MOD.

H&E staining showed that the tunica media of T2DM mice was thickened, which could be alleviated by vaccarin (Figure 3A). The mRNA and protein expressions of TNF-α and IL-1β in the aorta of T2DM mice increased significantly (Figures 3B, C, E–G), suggesting that the aorta of T2DM mice had an inflammatory injury. IHC staining displayed the same results (Figure 3I). Furthermore, the production of MDA increased while the activity of GSH-Px decreased (Figures 3J, K). However, these disorders were alleviated after vaccarin treatment (Figures 3B–K). It indicated that vaccarin had a remarkable effect on aortic inflammatory injury in T2DM mice. Beyond that, vaccarin could inhibit the abnormally elevated HDAC1 in the aorta of T2DM mice, which was consistent with the previous research results (Figures 3D, E, H).

### Vaccarin attenuated HG-induced inflammatory injury in HUVEC cells

**FIGURE 4:**
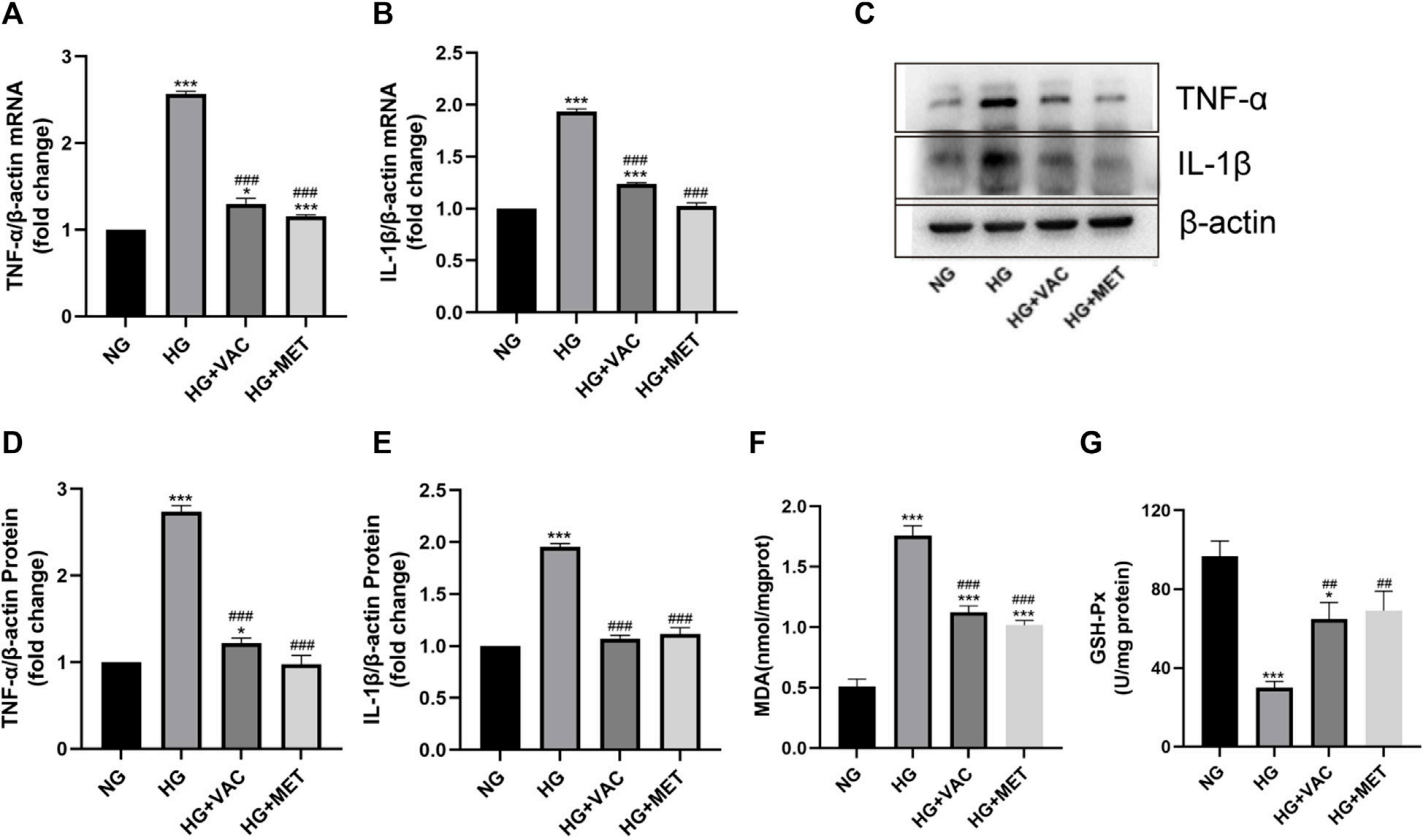
Vaccarin attenuated HG-induced inflammatory injury in HUVEC cells. **(A,B)**. The effects of vaccarin or metformin on the TNF-α mRNA and IL-1β mRNA expression in HG-induced HUVEC cells. **(C–E)**. Western blotting analysis of TNF-α and IL-1β protein expression levels in HG-induced HUVEC cells. Gray value of western blotting. **(F)**. Content of MDA. **(G)**. Activity of GSH-Px. Values are mean ± SEM. *n* = 3 in each group. *p < 0.05, **p < 0.01, ***p < 0.001 vs. NG; ^#^ *p* < 0.05, ^##^ *p* < 0.01, ^###^ *p* < 0.001 vs. HG.

TNF-α, IL-1β mRNA, and protein levels in HUVEC cells induced by HG increased significantly (Figures 4A–E). In addition, it was found that the production of MDA increased and the activity of GSH-Px decreased in HG-induced HUVEC cells, vaccarin treatment could reverse these phenomena (Figures 4F, G), indicating that vaccarin could also play an anti-inflammatory role *in vitro*. Metformin (MET) had been widely proven to relieve inflammatory injury in T2DM(Karam and Radwan 2019; Raj, Natarajan et al., 2021; Wang, Wang et al., 2022). Therefore, we used metformin as a positive control to compare the effects of vaccarin and found that the effect of vaccarin on the inflammatory injury was similar to that of metformin (Figures 4A–G). These phenomena aroused our interest and led us to further study the mechanism of vaccarin reducing HG-induced inflammatory injury.

### Vaccarin alleviated HG-induced inflammatory injury in HUVEC cells by inhibiting HDAC1 expression

**FIGURE 5:**
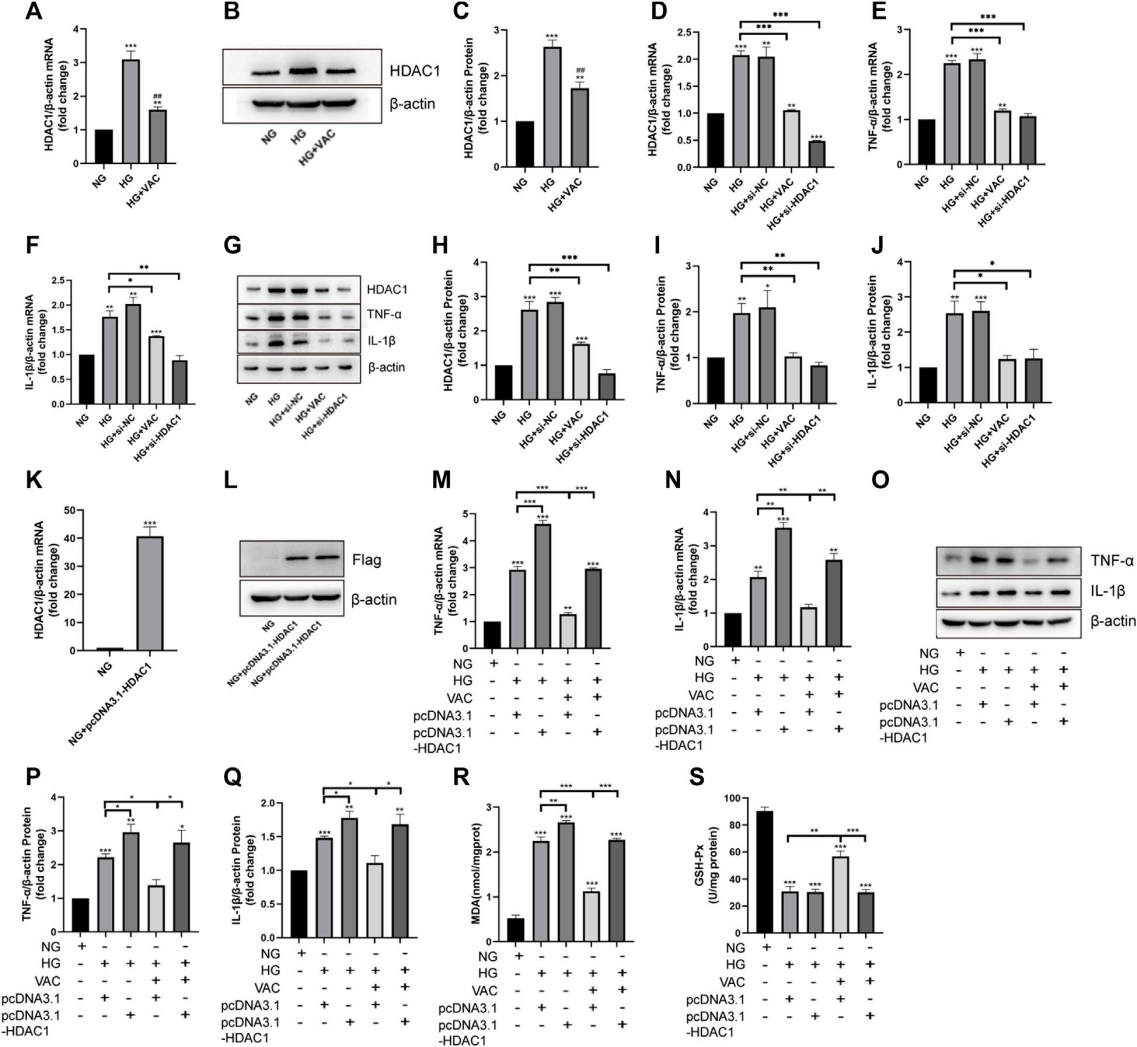
Vaccarin alleviated HG-induced inflammatory injury in HUVEC cells by inhibiting HDAC1 expression. **(A)**. The effects of vaccarin on HDAC1 mRNA expression in HG-induced HUVEC cells. **(B,C)**. Western blotting analysis of HDAC1 protein expression levels in HG-induced HUVEC cells. Gray value of western blotting. **(D–F)**. The expression of HDAC1 mRNA, TNF-α mRNA, and IL-1β mRNA in HUVEC cells transfected with siRNA NC or HDAC1 siRNA. **(G–J)**. Western blotting analysis of HDAC1, TNF-α, and IL-1β protein expression levels in HUVEC cells transfected with siRNA NC or HDAC1 siRNA. Gray value of western blotting. **(K)**. The expression of HDAC1 mRNA in HUVEC cells transfected with pcDNA3.1-HDAC1. **(L)**. Western blotting analysis of Flag protein expression levels in HUVEC cells transfected with pcDNA3.1-HDAC1. **(M,N)**. The effects of vaccarin on TNF-α mRNA and IL-1β mRNA expression in HUVEC cells transfected with pcDNA3.1 or pcDNA3.1-HDAC1. **(O–Q)**. Western blotting analysis of TNF-α and IL-1β protein expression levels in HUVEC cells transfected with pcDNA3.1 or pcDNA3.1-HDAC1. Gray value of western blotting. **(R)**. Content of MDA. **(S)**. Activity of GSH-Px. Values are mean ± SEM. n = 3 in each group. *p < 0.05, **p < 0.01, ***p < 0.001 vs. NG; ^#^ *p* < 0.05, ^##^ *p* < 0.01, ^###^ *p* < 0.001 vs. HG.

Consistent with the results of *in vivo* experiments, vaccarin could reverse the increased level of HDAC1 induced by HG *in vitro* (Figures 5A–C). To investigate the role of HDAC1 in HG-induced inflammatory injury, the silence of HDAC1 was made by transfection of HDAC1 siRNA (si-HDAC1). The effect of si-HDAC1 was similar to that of vaccarin, so there was reason to suspect that vaccarin functioned through HDAC1 (Figures 5D–J). Overexpression of HDAC1 was made by transfection of pcDNA3.1-HDAC1 (Figure 5K, L). We found that HDAC1 counteracted the inhibitory effects of vaccarin on TNF-α and IL-1β induced by HG in HUVEC cells (Figure 5M–Q). Meanwhile, the overexpression of HDAC1 also reversed the effects of vaccarin on mitigating MDA production and GSH-Px activity (Figure 5R, S). These results demonstrated that vaccarin alleviates the HG-induced inflammatory injury by decreasing the expression of HDAC1.

### Vaccarin inhibited the HG-induced decrease of miR-570-3p *in vitro*

**FIGURE 6:**
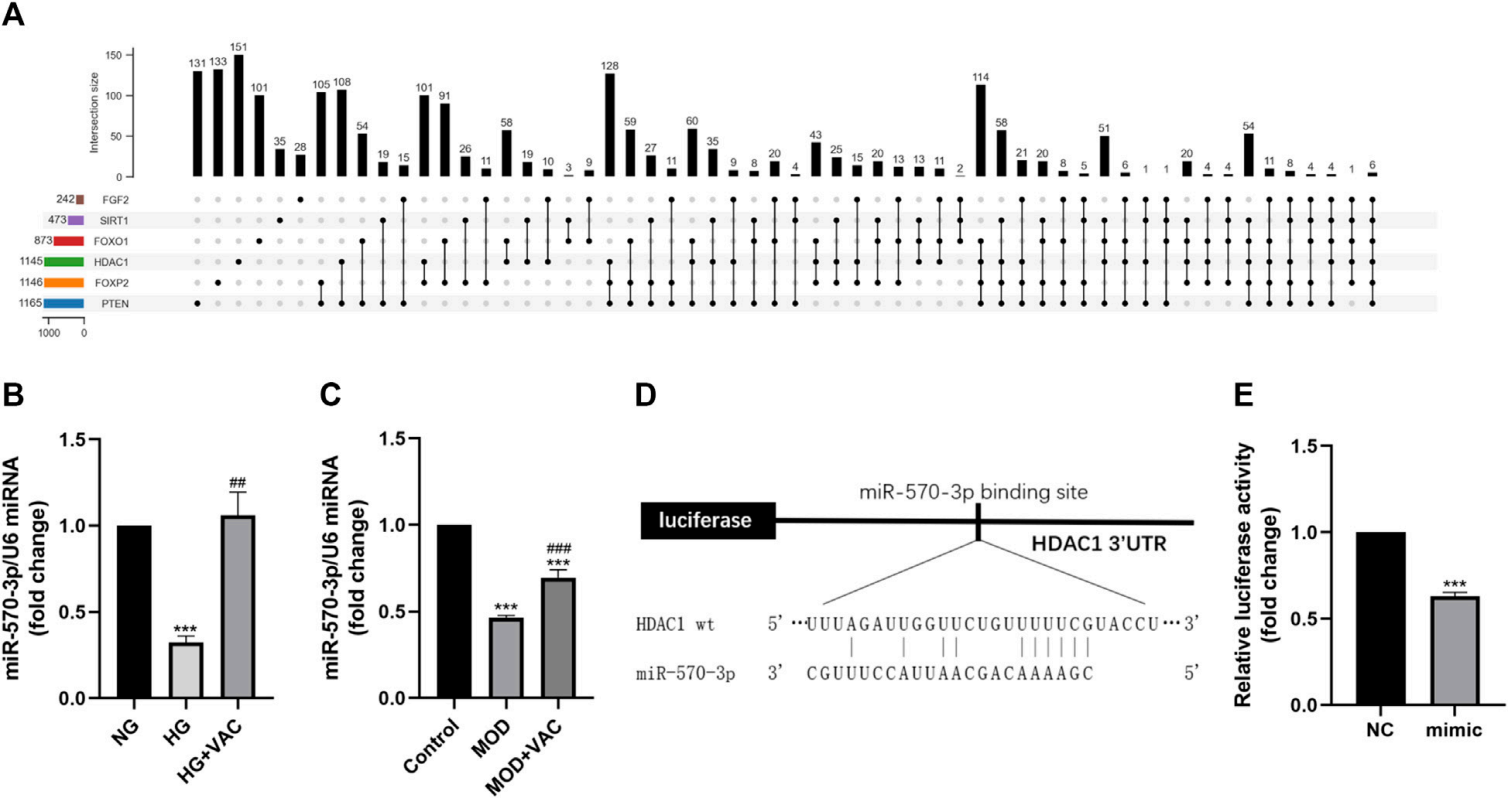
Vaccarin inhibited the HG-induced decrease of miR-570-3p *in vitro*. **(A)**. Upset graph is used to represent overlapping sets of elements for miRNAs. **(B–C)**. The effects of vaccarin on the expression of miR-570-3p miRNA in HG-induced HUVEC cells and aortic tissue. **(D)**. Complementary bases between miR-570-3p and the 3′ UTR of HDAC1. **(E)**. Luciferase activity was detected in the HUVEC cells. Values are mean ± SEM. n = 3 in each group. *p < 0.05, **p < 0.01, ***p < 0.001 vs. NG or Control; ^#^ *p* < 0.05, ^##^ *p* < 0.01, ^###^ *p* < 0.001 vs. HG or MOD.

Then we focused on miRNA to further inquire about the upstream regulatory of HDAC1 (Figure 6A). The results of RT-qPCR further confirmed that the level of miR-570-3p in HUVEC cells was suppressed in the HG group, while increasing after vaccarin treatment (Figure 6B). This trend was also proved in the aorta of T2DM mice (Figure 6C). The binding sites of HDAC1 and miR-570-3p were predicted by TargetScan (Figure 6D). The dual-luciferase reporter system was used to confirm the binding of miR-570-3p with the HDAC1 mRNA. Luciferase reporter vectors carrying the wild-type 3′-UTR of HDAC1 were transfected into HUVEC cells. It was observed that miR-570-3p mimic significantly suppressed the luciferase activity in the cells transfected with the luciferase vectors carrying the wild-type 3′-UTR of HDAC1, indicating that HDAC1 is a potential target of miR-570-3p (Figure 6E).

### Overexpression of miR-570-3p inhibited HDAC1 expression and inflammatory injury in HUVEC cells

**FIGURE 7:**
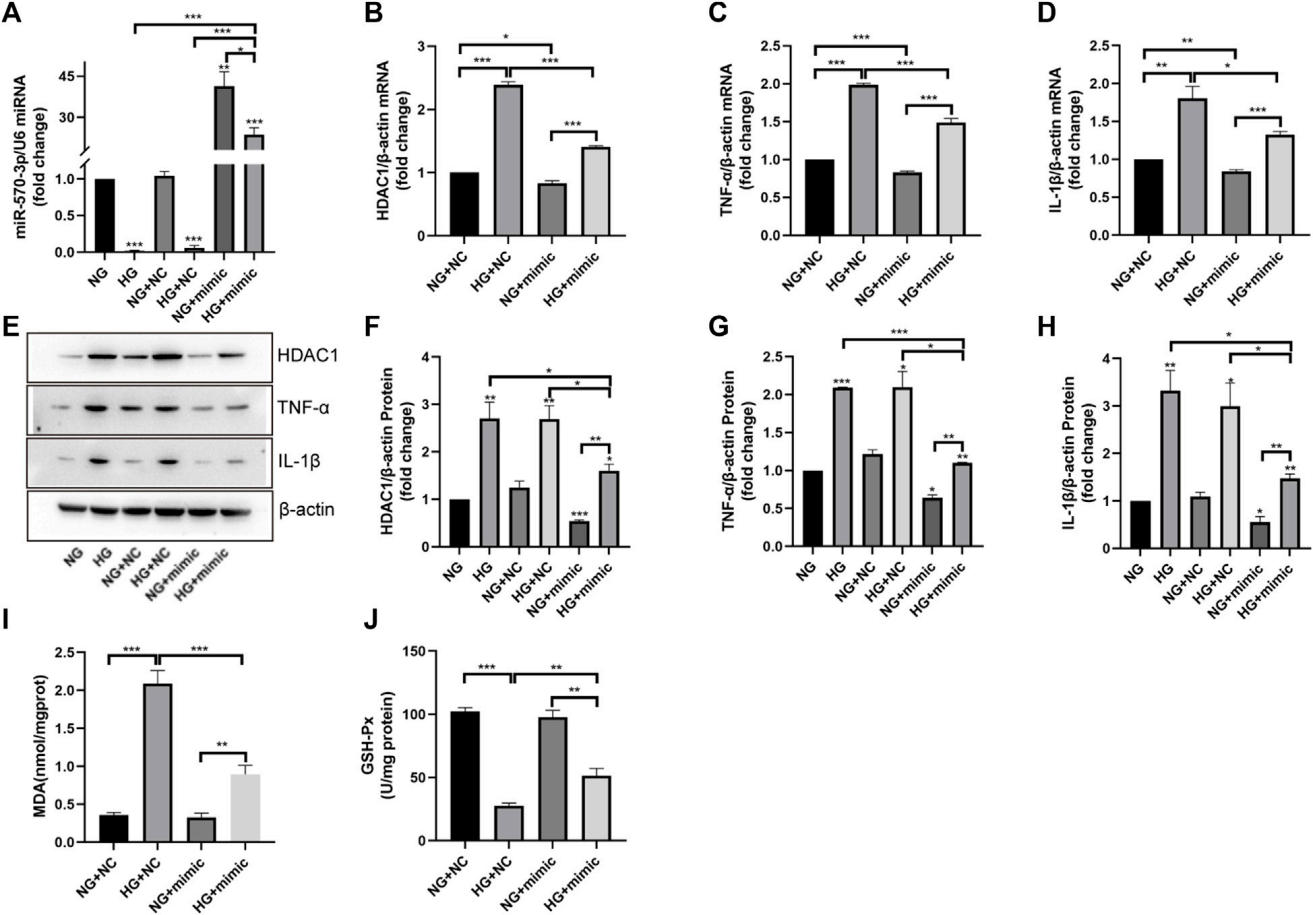
Overexpression of miR-570-3p inhibited HDAC1 expression and inflammatory injury in HUVEC cells. **(A)**. The expression of miR-570-3p miRNA in HUVEC cells transfected with miR-570-3p NC or miR-570-3p mimics. **(B–D)**. The expression of HDAC1 mRNA, TNF-α mRNA, and IL-1β mRNA in HUVEC cells transfected with miR-570-3p NC or miR-570-3p mimics. **(E–H)**. Western blotting analysis of HDAC1, TNF-α, and IL-1β protein expression levels in HUVEC cells transfected with miR-570-3p NC or miR-570-3p mimics. Gray value of western blotting. **(I)**. Content of MDA. **(J)**. Activity of GSH-Px. Values are mean ± SEM. n = 3 in each group. *p < 0.05, **p < 0.01, ***p < 0.001.

We further investigated the potential role of miR-570-3p in inflammatory injury. Through the application of miR-570-3p mimics, the expression of miR-570-3p in HUVEC cells was significantly increased (Figure 7A). The overexpression of miR-570-3p led to the inhibition of HDAC1 at the mRNA and protein levels (Figures 7B, E, F). Simultaneously, the application of miR-570-3p mimics also inhibited TNF-α and IL-1β, decreased MDA production, and increased GSH-Px activity (Figures 7C–E, G, J), thus alleviating the degree of inflammatory injury. From these phenomena, the overexpression of miR-570-3p had similar anti-inflammatory effects as vaccarin. Hence, we have reason to suspect that vaccarin plays an anti-inflammatory role through miR-570-3p.

### Vaccarin inhibited the expression of HDAC1 and inflammatory injury by up-regulating miR-570-3p

**FIGURE 8:**
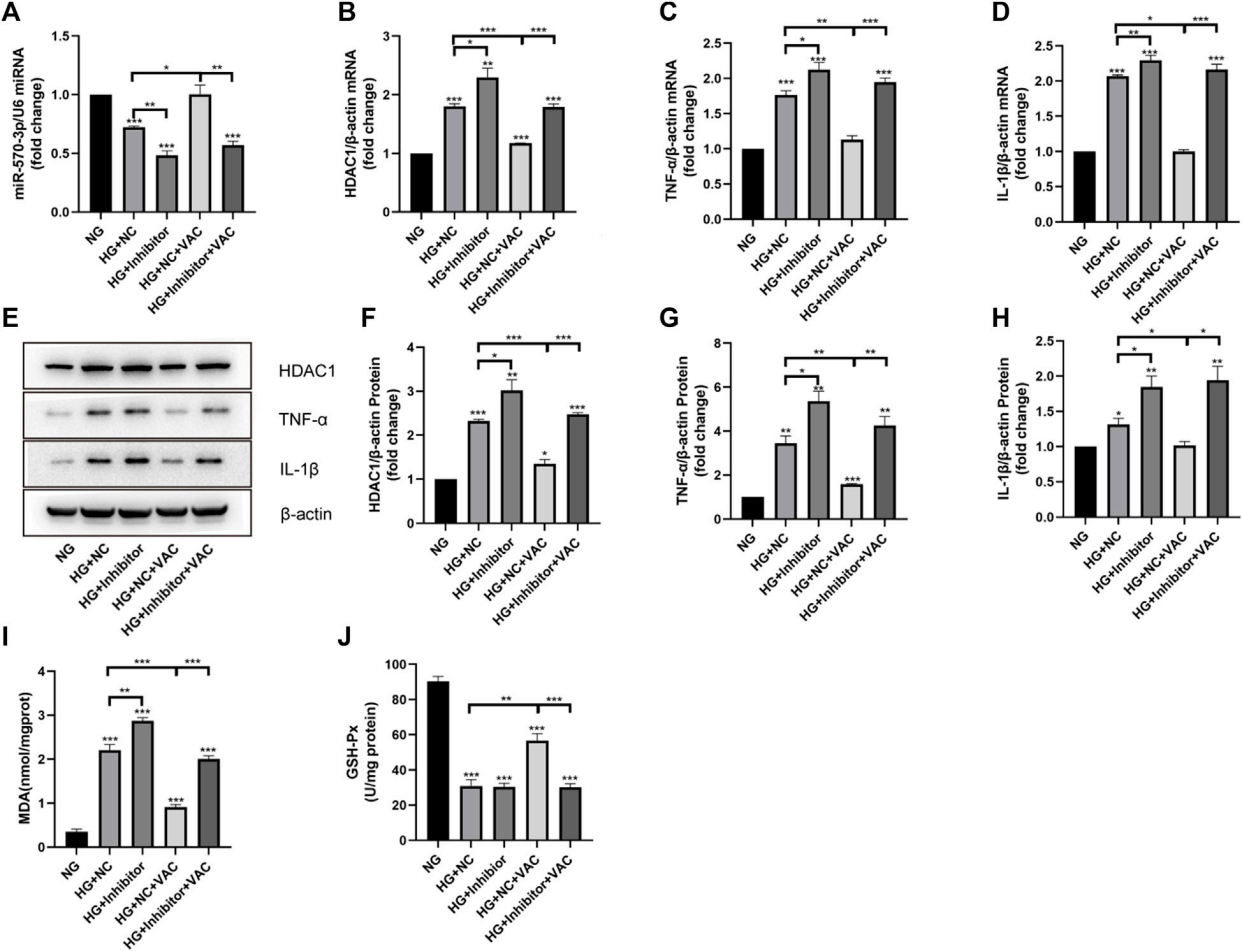
Vaccarin inhibited the expression of HDAC1 and inflammatory injury by up-regulating miR-570-3p. **(A)**. The effects of vaccarin on the expression of miR-570-3p miRNA in HUVEC cells transfected with miR-570-3p NC or miR-570-3p inhibitor. **(B–D)**. The effects of vaccarin on the expression of HDAC1 mRNA, TNF-α mRNA, and IL-1β mRNA in HUVEC cells transfected with miR-570-3p NC or miR-570-3p inhibitor. **(E–H)**. Western blotting analysis of HDAC1, TNF-α, and IL-1β protein expression levels in HUVEC cells transfected with miR-570-3p NC or miR-570-3p inhibitor. Gray value of western blotting. **(I)**. Content of MDA. **(J)**. Activity of GSH-Px. Values are mean ± SEM. *n* = 3 in each group. *p < 0.05, **p < 0.01, ***p < 0.001.

To determine whether vaccarin exerts anti-inflammatory effects through miR-570-3p, we transfected HUVEC cells with miR-570-3p inhibitor. The expression of miR-570-3p in HUVEC cells was significantly reduced after transfection, and the up-regulation of miR-570-3p by vaccarin was also observably offset (Figure 8A). In the meantime, the inhibition of vaccarin on HDAC1, TNF-α, and IL-1β was counteracted because of the application of miR-570-3p inhibitor (Figures 8B–H). It played a similar role in MDA production and GSH-Px activity (Figures 8I, J). It indicated that vaccarin will not play the role of anti-inflammatory in the absence of miR-570-3p, and proved that vaccarin can inhibit the expression of HDAC1 and inflammatory injury by upregulating miR-570-3p.

### Effect of vaccarin on the inflammatory injury when co-transfected with miR-570-3p mimics and pcDNA3.1-HDAC1

**FIGURE 9:**
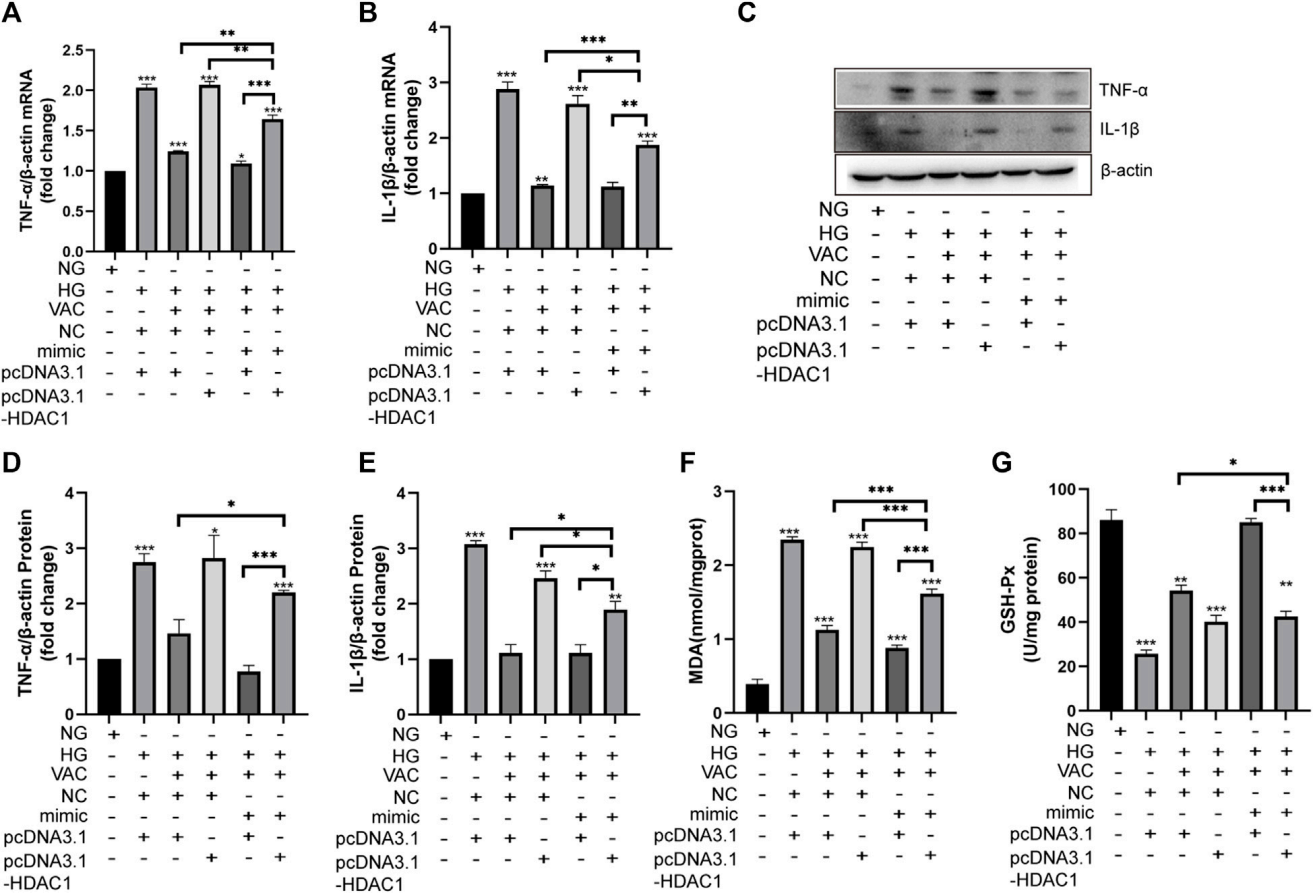
Effect of vaccarin on the inflammatory injury when co-transfected with miR-570-3p mimic and pcDNA3.1-HDAC1. **(A,B)**. The effects of vaccarin on the expression of TNF-α mRNA and IL-1β mRNA in HUVEC cells transfected with miR-570-3p NC or miR-570-3p mimics or pcDNA3.1 or pcDNA3.1-HDAC1. **(C,E)**. Western blotting analysis of TNF-α and IL-1β protein expression levels in HUVEC cells transfected with miR-570-3p NC or miR-570-3p mimics or pcDNA3.1 or pcDNA3.1-HDAC1. Gray value of western blotting. **(F)**. Content of MDA. **(G)**. Activity of GSH-Px. Values are mean ± SEM. n = 3 in each group. *p < 0.05, **p < 0.01, ***p < 0.001.

Finally, we studied the effects of vaccarin on inflammatory injury in HUVEC cells co-transfected with miR-570-3p mimics and pcDNA3.1-HDAC1. After co-transfection, the anti-inflammatory effects of vaccarin were inhibited, reversing the effect of vaccarin after transfection of miR-570-3p mimics alone. In contrast, the remission effect of vaccarin on inflammatory injury increased after co-transfection compared with pcDNA3.1-HDAC1 alone (Figures 9A–G).

## Discussion

Diabetic angiopathy develops in over half of diabetic patients, which is a severe threat to public health. Endothelial dysfunction is a critical early event in the pathogenesis of diabetic vascular complications (Jiang, Wu et al., 2020). Therefore, the improvement and treatment of endothelial dysfunction are of great significance for the prevention of vascular complications in patients with diabetes. Evidence has shown that hyperglycemia promotes inflammation and oxidative stress owing to endothelial dysfunction (Basta, Lazzerini et al., 2005; Hulsmans and Holvoet 2010; Mittal, Siddiqui et al., 2014; Sharma, Rizky et al., 2017). Thus, the improvement of anti-inflammation function may be beneficial to endothelial dysfunction. Recently, mounting attention has been paid to traditional Chinese medicines due to their therapeutic effects on diabetes and its complications such as macrovascular disease (Zhang, Xu et al., 2020). Vaccarin is an active monomer of saponaria vaccaria, an ancient Chinese medicine. Previous studies have proved that vaccarin shows the potential function in a variety of complications caused by diabetes, including macrovascular disease (Xie, Cai et al., 2015; Qiu, Du et al., 2016; Sun, Cai et al., 2017; Zhu, Lei et al., 2018; Lei, Gong et al., 2019; Xu, Liu et al., 2019; Hou, Cai et al., 2020; Hou, Qi et al., 2020; Liu, Sun et al., 2020; Sun, Yu et al., 2021). However, the effect of vaccarin on alleviating endothelial inflammatory injury in T2DM and the molecular mechanism has not been elucidated. In this study, we aim to further investigate the effect of vaccarin on vascular disease in T2DM and to further clarify its pharmacological effects.

First of all, vaccarin effectively reduced the blood glucose of T2DM mice (Figures 2C, D), improved glucose tolerance (Figures 2E, F), insulin tolerance (Figures 2G, H), a disorder of glucose and lipid metabolism, and physiological conditions of T2DM mice (Figures 2I–M), and also had a good effect on alleviating the inflammatory injury in the aorta of T2DM mice (Figures 3B–K). In the HG-induced HUVEC cells, vaccarin had a similar effect *in vitro* (Figures 4A–G).

Histone deacetylases (HDACs) remove histone acetylation marks, resulting in compaction of chromatin structure and transcriptional repression. HDACs operate by direct association with DNA binding factors, and by incorporation into large multifunctional repressor complexes. HDACs form a large family, of which Class I HDACs(LeBoeuf, Terrell et al., 2010), including HDAC1, show the strongest histone deacetylase activity (Haberland, Montgomery et al., 2009; LeBoeuf, Terrell et al., 2010). There are relevant studies on the relationship between HDAC1 and inflammation. For example, down-regulation of HDAC1 can restore myocardial injury in septic mice to a certain extent (Nong, Qin et al., 2022), and ellagic acid can alleviate rheumatoid arthritis in rats by inhibiting HDAC1 (Song, Wu et al., 2021), and HDAC1 can control the metabolism of intestinal epithelial cells by regulating the supply of acetyl groups, resulting in impaired response to oxidative stress, AMPK kinase activation and mitochondrial biogenesis (Gonneaud, Turgeon et al., 2015). Our study found that the expression of HDAC1 was significantly increased in the aorta of T2DM mice as well as HG-induced HUVEC cells (Figures 3D, E, H, Figures 5A–C). Supplementary vaccarin or HDAC1 siRNA inhibited the expression of HDAC1 and alleviated inflammatory injury (Figures 5A–J). Overexpression of HDAC1 had offset the inhibitory effects of vaccarin on inflammatory injury in HG-induced HUVEC cells (Figure 5M–S). These results indicated that vaccarin suppressed inflammatory injury by inhibiting HDAC1.

In recent years, the role of miRNA in biological physiological, and pathological processes has gradually become clear, especially in the field of chronic diseases such as diabetes. Studies have found that miRNA-34a, miRNA-24, miRNA-181c, miRNA-29b, miRNA-200a, miRNA-383, and other miRNAs play a role in inflammation (Li, Kim et al., 2016; Lo, Yang et al., 2018; Shen, Li et al., 2018; Cheng, Chen et al., 2019; Zhang, Cai et al., 2019; Hu, Gong et al., 2020), thus participating in the occurrence of endothelial dysfunction in diabetes. To study the exact mechanism of vaccarin in regulating HDAC1, databases were used to screen the possible miRNA targets (Figure 6A). We found that the 3′ UTR of HDAC1’s mRNA was a potential target of miR-570-3p which declined in the aorta of T2DM mice and HG-induced HUVEC cells, while increased with supplementary vaccarin (Figures 6B, C). The binding of miR-570-3p with the 3′ UTR of HDAC1’s mRNA was confirmed by the dual-luciferase reporter system (Figures 6D, E). From a phenomenal point of view, miR-570-3p mimic and vaccarin had similar effects on inflammatory injury (Figures 7A–J). Giving miR-570-3p inhibitor offset the inhibition of vaccarin on HDAC1 and inflammatory injury in HUVEC cells (Figures 8A–J). Finally, we co-transfected miR-570-3p mimic and pcDNA3.1-HDAC1 to study the role of vaccarin in this case (Figures 9A–G). These results indicated that vaccarin alleviates diabetic inflammatory injury by mediating the miR-570-3p/HDAC1 pathway.

**FIGURE 10:**
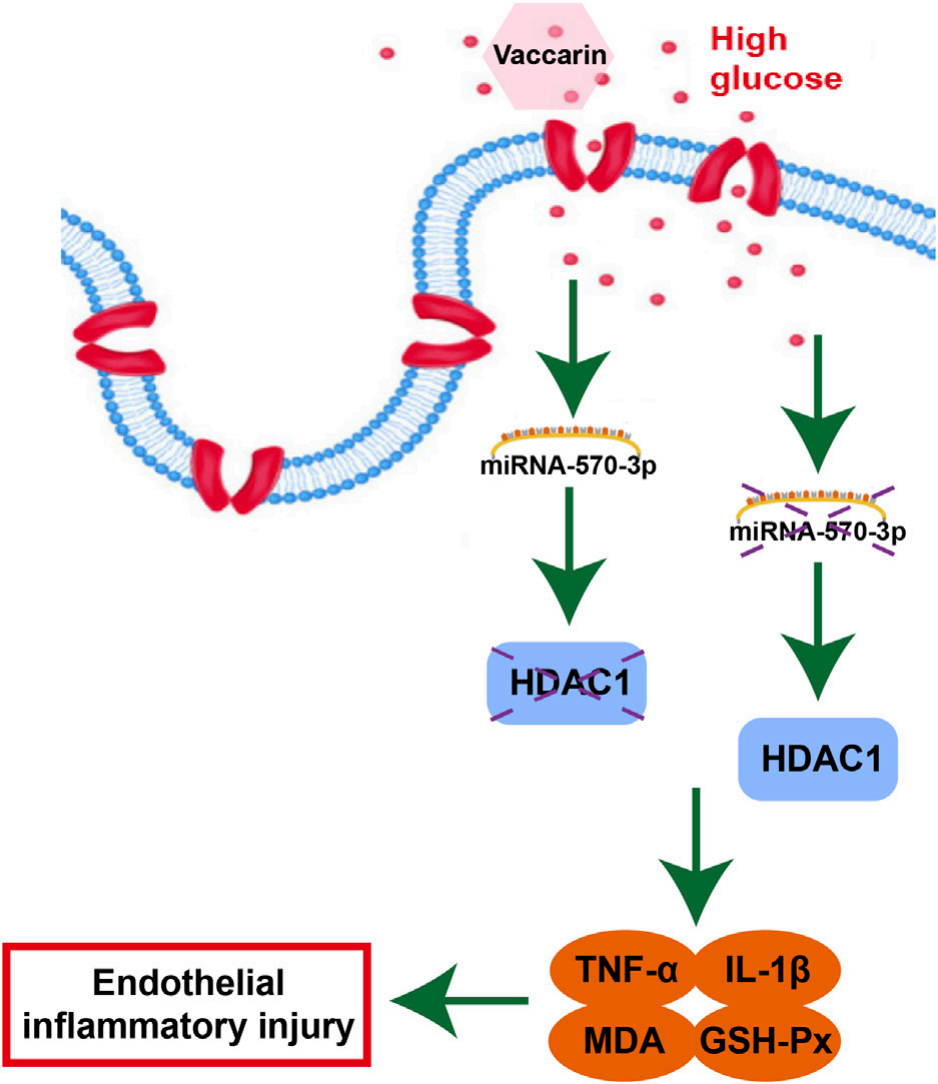
Protective mechanisms of vaccarin against HG-induced endothelial inflammatory injury.

In brief, vaccarin alleviates inflammatory injury by restoring miR-570-3p/HDAC1 in HUVEC cells stimulated by HG. Based on these results, we propose a novel mechanism by which vaccarin protects endothelial function, which may provide a novel treatment strategy for vascular complications in T2DM. Our findings provide new insights into the protective role of vaccarin in diabetic endothelial dysfunction, suggesting that miR-570-3p and HDAC1 may serve as biomarkers of vascular endothelial inflammatory injury and potential therapeutic targets. We provide evidence that under diabetic conditions, overexpression of miR-570-3p alleviates endothelial dysfunction by reducing HDAC1 expression and endothelial inflammatory injury, thereby reducing endothelial dysfunction. In conclusion, our outcomes show that vaccarin can reduce inflammatory injury by upregulating miR-570-3p thus inhibiting the increase of HDAC1 in the aorta of T2DM mice and HG-treated HUVEC cells (Figure 10), which provides new ideas, insights, and choices for the scope of application and medicinal value of vaccarin and some potential biomarkers or targets in diabetic endothelial dysfunction and vascular complications. Of course, this experiment also has shortcomings. We need to achieve the inhibition and overexpression of miR-570-3p and HDAC1 *in vivo* in order to further verify these conclusions.

## Data Availability

The original contributions presented in the study are included in the article/supplementary material further inquiries can be directed to the corresponding authors.

## References

[B1] Ai M. Li S. S. Chen H. Wang X. T. Sun J. N. Hou B. (2021). 1, 25(OH)2 D3 attenuates sleep disturbance in mouse models of Lewis lung cancer, *in silico* and *in vivo* . J. Cell. Physiol. 236 (11), 7473–7490. 10.1002/jcp.30458 34061988

[B2] Baker J. R. Vuppusetty C. Colley T. Hassibi S. Fenwick P. S. Donnelly L. E. (2018). MicroRNA‐570 is a novel regulator of cellular senescence and inflammaging. FASEB J. 33 (2), 1605–1616. 10.1096/fj.201800965R 30156909 PMC6338629

[B3] Basta G. Lazzerini G. Del Turco S. Ratto G. M. Schmidt A. M. De Caterina R. (2005). At least 2 distinct pathways generating reactive oxygen species mediate vascular cell adhesion molecule-1 induction by advanced glycation end products. Arterioscler. Thromb. Vasc. Biol. 25 (7), 1401–1407. 10.1161/01.ATV.0000167522.48370.5e 15845907

[B4] Betel D. Koppal A. Agius P. Sander C. Leslie C. (2010). Comprehensive modeling of microRNA targets predicts functional non-conserved and non-canonical sites. Genome Biol. 11 (8), R90. 10.1186/gb-2010-11-8-r90 20799968 PMC2945792

[B5] Booton R. Lindsay M. A. (2014). Emerging role of MicroRNAs and long noncoding RNAs in respiratory disease. Chest 146 (1), 193–204. 10.1378/chest.13-2736 25010962

[B6] Cheng X. W. Chen Z. F. Wan Y. F. Zhou Q. Wang H. Zhu H. Q. (2019). Long non-coding RNA H19 suppression protects the endothelium against hyperglycemic-induced inflammation via inhibiting expression of miR-29b target gene vascular endothelial growth factor a through activation of the protein kinase B/endothelial nitric oxide synthase pathway. Front. Cell Dev. Biol. 7, 263. 10.3389/fcell.2019.00263 31737629 PMC6838022

[B7] Dhananjayan R. Koundinya K. S. Malati T. Kutala V. K. (2016). Endothelial dysfunction in type 2 diabetes mellitus. Indian J. Clin. biochem. 31 (4), 372–379. 10.1007/s12291-015-0516-y 27605734 PMC4992481

[B8] 2014). Diagnosis and classification of diabetes mellitus. Diabetes Care 37 (1), S81–S90. 10.2337/dc14-s081 24357215

[B9] Gonneaud A. Turgeon N. Boisvert F. M. Boudreau F. Asselin C. (2015). Loss of histone deacetylase Hdac1 disrupts metabolic processes in intestinal epithelial cells. FEBS Lett. 589 (19), 2776–2783. 10.1016/j.febslet.2015.08.009 26297832

[B10] Haberland M. Montgomery R. L. Olson E. N. (2009). The many roles of histone deacetylases in development and physiology: Implications for disease and therapy. Nat. Rev. Genet. 10 (1), 32–42. 10.1038/nrg2485 19065135 PMC3215088

[B11] Hou B. Cai W. Chen T. Zhang Z. Gong H. Yang W. (2020). Vaccarin hastens wound healing by promoting angiogenesis via activation of MAPK/ERK and PI3K/AKT signaling pathways *in vivo* . Acta Cir. Bras. 34 (12), e201901202. 10.1590/s0102-865020190120000002 32049183 PMC7006371

[B12] Hou B. Qi M. Sun J. Ai M. Ma X. Cai W. (2020). Preparation, characterization and wound healing effect of vaccarin-chitosan nanoparticles. Int. J. Biol. Macromol. 165, 3169–3179. 10.1016/j.ijbiomac.2020.10.182 33122060

[B13] Hu B. Gong Z. Bi Z. (2020). Inhibition of miR-383 suppresses oxidative stress and improves endothelial function by increasing sirtuin 1. Braz J. Med. Biol. Res. 53 (2), e8616. 10.1590/1414-431X20198616 31994599 PMC6984384

[B14] Hulsmans M. Holvoet P. (2010). The vicious circle between oxidative stress and inflammation in atherosclerosis. J. Cell. Mol. Med. 14 (1-2), 70–78. 10.1111/j.1582-4934.2009.00978.x 19968738 PMC3837590

[B15] Jiang Z. Wu J. Ma F. Jiang J. Xu L. Du L. (2020). MicroRNA-200a improves diabetic endothelial dysfunction by targeting KEAP1/NRF2. J. Endocrinol. 245 (1), 129–140. 10.1530/JOE-19-0414 32031966

[B16] Kannel W. B. McGee D. L. (1979). Diabetes and cardiovascular disease. The Framingham study. JAMA 241 (19), 2035–2038. 10.1001/jama.241.19.2035 430798

[B17] Karam H. M. Radwan R. R. (2019). Metformin modulates cardiac endothelial dysfunction, oxidative stress and inflammation in irradiated rats: A new perspective of an antidiabetic drug. Clin. Exp. Pharmacol. Physiol. 46 (12), 1124–1132. 10.1111/1440-1681.13148 31357226

[B18] LeBoeuf M. Terrell A. Trivedi S. Sinha S. Epstein J. A. Olson E. N. (2010). Hdac1 and Hdac2 act redundantly to control p63 and p53 functions in epidermal progenitor cells. Dev. Cell 19 (6), 807–818. 10.1016/j.devcel.2010.10.015 21093383 PMC3003338

[B19] Lei Y. Gong L. Tan F. Liu Y. Li S. Shen H. (2019). Vaccarin ameliorates insulin resistance and steatosis by activating the AMPK signaling pathway. Eur. J. Pharmacol. 851, 13–24. 10.1016/j.ejphar.2019.02.029 30779918

[B20] Li Q. Kim Y. R. Vikram A. Kumar S. Kassan M. Gabani M. (2016). P66Shc-Induced MicroRNA-34a causes diabetic endothelial dysfunction by downregulating Sirtuin1. Arterioscler. Thromb. Vasc. Biol. 36 (12), 2394–2403. 10.1161/ATVBAHA.116.308321 27789474 PMC5293179

[B21] Liu Y. Sun J. Ma X. Li S. Ai M. Xu F. (2020). Vaccarin regulates diabetic chronic wound healing through FOXP2/AGGF1 pathways. Int. J. Mol. Sci. 21 (6), E1966. 10.3390/ijms21061966 32183046 PMC7139532

[B22] Lo W. Y. Yang W. K. Peng C. T. Pai W. Y. Wang H. J. (2018). MicroRNA-200a/200b modulate high glucose-induced endothelial inflammation by targeting O-linked N-acetylglucosamine transferase expression. Front. Physiol. 9, 355. 10.3389/fphys.2018.00355 29720943 PMC5915961

[B23] Mittal M. Siddiqui M. R. Tran K. Reddy S. P. Malik A. B. (2014). Reactive oxygen species in inflammation and tissue injury. Antioxid. Redox Signal. 20 (7), 1126–1167. 10.1089/ars.2012.5149 23991888 PMC3929010

[B24] Nong R. Qin C. Lin Q. Lu Y. Li J. (2022). Down-regulated HDAC1 and up-regulated microRNA-124-5p recover myocardial damage of septic mice. Bioengineered 13 (3), 7168–7180. 10.1080/21655979.2022.2034583 35285407 PMC9278975

[B25] Ostergard T. Nyholm B. Hansen T. K. Rasmussen L. M. Ingerslev J. Sorensen K. E. (2006). Endothelial function and biochemical vascular markers in first-degree relatives of type 2 diabetic patients: The effect of exercise training. Metabolism. 55 (11), 1508–1515. 10.1016/j.metabol.2006.06.024 17046554

[B26] Qiu Y. Du B. Xie F. Cai W. Liu Y. Li Y. (2016). Vaccarin attenuates high glucose-induced human EA*hy926 endothelial cell injury through inhibition of Notch signaling. Mol. Med. Rep. 13 (3), 2143–2150. 10.3892/mmr.2016.4801 26795539

[B27] Raj V. Natarajan S. Chatterjee S. Ramasamy M. Ramanujam G. M. Arasu M. V. (2021). Cholecalciferol and metformin protect against lipopolysaccharide-induced endothelial dysfunction and senescence by modulating sirtuin-1 and protein arginine methyltransferase-1. Eur. J. Pharmacol. 912, 174531. 10.1016/j.ejphar.2021.174531 34710370

[B28] Roberts A. C. Porter K. E. (2013). Cellular and molecular mechanisms of endothelial dysfunction in diabetes. Diab. Vasc. Dis. Res. 10 (6), 472–482. 10.1177/1479164113500680 24002671

[B29] Sharma A. Rizky L. Stefanovic N. Tate M. Ritchie R. H. Ward K. W. (2017). The nuclear factor (erythroid-derived 2)-like 2 (Nrf2) activator dh404 protects against diabetes-induced endothelial dysfunction. Cardiovasc. Diabetol. 16 (1), 33. 10.1186/s12933-017-0513-y 28253885 PMC5335831

[B30] Shen X. Li Y. Sun G. Guo D. Bai X. (2018). miR-181c-3p and -5p promotes high-glucose-induced dysfunction in human umbilical vein endothelial cells by regulating leukemia inhibitory factor. Int. J. Biol. Macromol. 115, 509–517. 10.1016/j.ijbiomac.2018.03.173 29605252

[B31] Song H. Wu H. Dong J. Huang S. Ye J. Liu R. (2021). Ellagic acid alleviates rheumatoid arthritis in rats through inhibiting MTA1/HDAC1-mediated Nur77 deacetylation. Mediat. Inflamm. 2021, 6359652. 10.1155/2021/6359652 PMC8677414 34924813

[B32] Sun H. J. Cai W. W. Gong L. L. Wang X. Zhu X. X. Wan M. Y. (2017). FGF-2-mediated FGFR1 signaling in human microvascular endothelial cells is activated by vaccarin to promote angiogenesis. Biomed. Pharmacother. 95, 144–152. 10.1016/j.biopha.2017.08.059 28841454

[B33] Sun J. N. Yu X. Y. Hou B. Ai M. Qi M. T. Ma X. Y. (2021). Vaccarin enhances intestinal barrier function in type 2 diabetic mice. Eur. J. Pharmacol. 908, 174375. 10.1016/j.ejphar.2021.174375 34303666

[B34] Tan Y. Jiang C. Jia Q. Wang J. Huang G. Tang F. (2022). A novel oncogenic seRNA promotes nasopharyngeal carcinoma metastasis. Cell Death Dis. 13 (4), 401. 10.1038/s41419-022-04846-1 35461306 PMC9035166

[B35] Tang S.-t. Wang F. Shao M. Wang Y. Zhu H.-q. (2017). MicroRNA-126 suppresses inflammation in endothelial cells under hyperglycemic condition by targeting HMGB1. Vasc. Pharmacol. 88, 48–55. 10.1016/j.vph.2016.12.002 27993686

[B36] Vanhoutte P. M. Shimokawa H. Feletou M. Tang E. H. (2017). Endothelial dysfunction and vascular disease - a 30th anniversary update. Acta Physiol. 219 (1), 22–96. 10.1111/apha.12646 26706498

[B37] Wang G. Wang Y. Yang Q. Xu C. Zheng Y. Wang L. (2022). Metformin prevents methylglyoxal-induced apoptosis by suppressing oxidative stress *in vitro* and *in vivo* . Cell Death Dis. 13 (1), 29. 10.1038/s41419-021-04478-x 35013107 PMC8748764

[B38] Wang J. Liu D. Liang X. Gao L. Yue X. Yang Y. (2013). Construction of a recombinant eukaryotic human ZHX1 gene expression plasmid and the role of ZHX1 in hepatocellular carcinoma. Mol. Med. Rep. 8 (5), 1531–1536. 10.3892/mmr.2013.1700 24064680

[B39] Wu J. Jiang Z. Zhang H. Liang W. Huang W. Zhang H. (2018). Sodium butyrate attenuates diabetes-induced aortic endothelial dysfunction via P300-mediated transcriptional activation of Nrf2. Free Radic. Biol. Med. 124, 454–465. 10.1016/j.freeradbiomed.2018.06.034 29964168

[B40] Xie F. Cai W. Liu Y. Li Y. Du B. Feng L. (2015). Vaccarin attenuates the human EA.hy926 endothelial cell oxidative stress injury through inhibition of Notch signaling. Int. J. Mol. Med. 35 (1), 135–142. 10.3892/ijmm.2014.1977 25352009

[B41] Xu F. Liu Y. Zhu X. Li S. Shi X. Li Z. (2019). Protective effects and mechanisms of vaccarin on vascular endothelial dysfunction in diabetic angiopathy. Int. J. Mol. Sci. 20 (18), E4587. 10.3390/ijms20184587 31533227 PMC6769517

[B42] Yi J. Gao Z. F. (2019). MicroRNA-9-5p promotes angiogenesis but inhibits apoptosis and inflammation of high glucose-induced injury in human umbilical vascular endothelial cells by targeting CXCR4. Int. J. Biol. Macromol. 130, 1–9. 10.1016/j.ijbiomac.2019.02.003 30716366

[B43] Zhang J. Cai W. Fan Z. Yang C. Wang W. Xiong M. (2019). MicroRNA-24 inhibits the oxidative stress induced by vascular injury by activating the Nrf2/Ho-1 signaling pathway. Atherosclerosis 290, 9–18. 10.1016/j.atherosclerosis.2019.08.023 31539718

[B44] Zhang S. Xu L. Liang R. Yang C. Wang P. (2020). Baicalin suppresses renal fibrosis through microRNA-124/TLR4/NF-κB axis in streptozotocin-induced diabetic nephropathy mice and high glucose-treated human proximal tubule epithelial cells. J. Physiol. Biochem. 76 (3), 407–416. 10.1007/s13105-020-00747-z 32500512

[B45] Zhu X. Lei Y. Tan F. Gong L. Gong H. Yang W. (2018). Vaccarin protects human microvascular endothelial cells from apoptosis via attenuation of HDAC1 and oxidative stress. Eur. J. Pharmacol. 818, 371–380. 10.1016/j.ejphar.2017.09.052 29128366

